# CSI-Former: Pay More Attention to Pose Estimation with WiFi

**DOI:** 10.3390/e25010020

**Published:** 2022-12-22

**Authors:** Yue Zhou, Caojie Xu, Lu Zhao, Aichun Zhu, Fangqiang Hu, Yifeng Li

**Affiliations:** School of Computer Science and Technology, Nanjing Tech University, Nanjing 211816, China

**Keywords:** pose estimation, multi-head attention, CSI, WiFi

## Abstract

Cross-modal human pose estimation has a wide range of applications. Traditional image-based pose estimation will not work well in poor light or darkness. Therefore, some sensors such as LiDAR or Radio Frequency (RF) signals are now using to estimate human pose. However, it limits the application that these methods require much high-priced professional equipment. To address these challenges, we propose a new WiFi-based pose estimation method. Based on the Channel State Information (CSI) of WiFi, a novel architecture CSI-former is proposed to innovatively realize the integration of the multi-head attention in the WiFi-based pose estimation network. To evaluate the performance of CSI-former, we establish a span-new dataset Wi-Pose. This dataset consists of 5 GHz WiFi CSI, the corresponding images, and skeleton point annotations. The experimental results on Wi-Pose demonstrate that CSI-former can significantly improve the performance in wireless pose estimation and achieve more remarkable performance over traditional image-based pose estimation. To better benefit future research on the WiFi-based pose estimation, Wi-Pose has been made publicly available.

## 1. Introduction

Pose estimation is a basic task of human behavior evaluation. It aims to analyze important joints of human bodies such as limbs and faces through a series of multimedia signals, and promote the recognition of human behaviors [1,2,3,4,5,6,7,8,9,10]. Nevertheless, traditional image-based pose estimation methods [11,12,13,14,15,16,17,18,19,20] usually do not work well under partial occlusion and poor lighting conditions. For example, the camera cannot collect clear and easy-to-estimate human images in glare interference or complete darkness. In addition, public concerns about privacy issues also limit the application of image-based pose estimation. Thus, it is urgent to find a cross-modal method to realize pose estimation when it is hard to capture human images.

In previous exploratory work, people have tried human pose estimation based on multiple signals. Adib et al. [21] try to use radio signals to locate human bodies. However, this method can only perform coarse-grained positioning analysis of human bodies, not fine-grained behavior analysis. Another example is the use of radiofrequency [22] signals for positioning and poses estimation of human bodies. However, this method requires the establishment of a special radio frequency signal equipment that is uncommon in daily life. Therefore, the high cost and the strict requirements for the installation environment of this method limit its application.

Compared with the above sensors, WiFi equipment has the advantages of lower costs and easy-to-layout. Therefore, using WiFi equipment for pose estimation is a good choice. Traditional WiF devices work under the wireless transmission standard protocol of the IEEE 802.11n [23]. Under the principle that human bodies consist of hierarchical organizations with different dielectric parameters [24,25,26], it receives changing WiFi data packet information through changing body poses in the WiFi signal field. Then, the received wireless information is parsed into Channel State Information (CSI) with 30 sub-carrier groups according to Orthogonal Frequency Division Multiplexing (OFDM) and serves as the original input of the network. In past research, people were constantly trying to build better neural network models to extract more significant feature information from CSI [27,28,29,30,31,32,33]. The work of Hao et al. [30] realized CSI-based gesture estimation. They tried to find those features that are more sensitive to gesture actions from CSI, and successfully established a well-performed gesture estimation model.

Motivated by the work of Hao et al. [30], we attempt to seek a reasonable method to extract pose features from the sub-carries that are more sensitive to most poses. However, the previous work [34] takes a 16-layers Resnet as the pose feature extractor and equally extracts the pose feature on the 30 sub-carriers of CSI. It indicates that the network cannot pay more attention to the information-rich sub-carries. Of course, this limits the performance of the network. Recently, Transformer [35] has shown impressive performance in various natural language processing [36,37] and computer vision tasks [38,39,40,41,42], due to its powerful multi-head attention. Following the attention mechanism of Transformer, a new Performer [43] is proposed to realize attention-based long sequence data analysis such as protein modeling. Benefit from its Fast Attention Via positive Orthogonal Random features approach (FAVOR+), Performer can linearly optimize traditional softmax attention calculation. It indicates that Performer has an excellent attention mechanism and better space utilization over traditional Transformer. Motivated by these advantages of Performer, we attempt to incorporate an attention mechanism to extract more hidden pose features from long sequence CSI. In addition, compared with traditional methods, we are committed to making the information-rich sub-carries receive more attention.

Consequently, this paper addresses the above problems and attempts to design a novel network for paying more attention to the information-rich sub-carries. To achieve this, a novel multi-head attention-based network CSI-former is proposed for WiFi-based pose estimation. The proposed CSI-former is built in a teacher–student manner: a teacher network based on Alphapose and a student network based on Performer [43] and convolutional neural network. The Alphapose-based teacher network is developed to generate skeleton point annotation of the input human images. The skeleton point annotation is used as the ground truth of the human poses to optimize the student network. In the student network, an attention-based Encoder-Decoder structure is proposed to extract pose feature from CSI and match it with the pose labels from the teacher network. Under the supervision of the teacher network, the student network keeps self-optimized learning until it realizes pure CSI-based pose estimation.

To demonstrate the performance of CSI-former, a reasonable and persuasive WiFi-based dataset is necessary. As the basis of establishing the dataset, it is significant for us to determine the WiFi frequency band. Most traditional CSI collection methods rely on a 2.4 GHz WiFi band. Nevertheless, the research of Yu et al. [44] demonstrates that compared with 2.4 GHz, the 5 GHz signals have strong anti-interference and fewer interference sources. Thus, we regard that 5 GHz WiFi is more suitable for a WiFi-based pose estimation network. Based on this, 12 volunteers were invited to perform indoor activities while collecting their images and CSI. Thus, a new WiFi-based pose estimation dataset named Wi-Pose that is composed of images, skeleton point annotations, and CSI was constructed. To the best of our knowledge, there is no such public dataset yet. The experimental results on Wi-Pose demonstrate that CSI-former achieves excellent performance on WiFi-based human pose estimation.

The contributions of this paper are summarized as follows:

1. We propose a novel architecture CSI-former that is composed of the attention mechanism and traditional convolutional neural network for WiFi-based pose estimation. To the best of our knowledge, CSI-former firstly realizes the effective integration of the multi-head attention to the field of cross-modal human pose estimation and significantly improves the performance.
2. Differ from most previous methods, we establish the WiFi-based human body pose estimation dataset via a 5 GHz wireless WiFi signal and successfully demonstrate the effectiveness of 5 GHz WiFi in cross-modal pose estimation.
3. We successfully established the novel WiFi-based pose estimation dataset Wi-Pose that is composed of 12 different actions such as bending, circling, crouching, pulling, running, walking, waving, etc. In addition, to ensure the rationality and persuasiveness of Wi-Pose, the invited 12 volunteers have different heights and weights.
4. The proposed CSI-former has been evaluated on Wi-Pose and compared with the traditional network. The results demonstrate that CSI-former achieves state-of-the-art performance in WiFi-based pose estimation.

In summary, we propose a WiFi-based pose estimation algorithm CSI-former in this paper to solve the performance defects and privacy problems of traditional image-based human pose estimation methods. Moreover, we present the attention mechanism-based network framework to improve the algorithm and achieve satisfactory performance. We also publish the new dataset Wi-Pose at https://github.com/NjtechCVLab/Wi-PoseDataset, accessed on 19 October 2022 to facilitate future research.

The rest of the article is organized as follows. We introduce the related work for human pose estimation in Section 2. Section 3 describes the proposed CSI-former in detail. In Section 4, we introduce the details and results of the experiment. Finally, we conclude in Section 5 by highlighting the innovation of our work and discussing the future work.

## 2. Related Work

### 2.1. Video-Based Human Pose Estimation

Liu et al. [45] proposed a two-stream convolutional neural network architecture with a spatiotemporal network. The network uses the camera to capture videos for human body pose estimation. Each video is divided into two parts: a space part for describing static information of the scene and the object, a time part for describing motion information of the object and the camera. However, since the access of the two-stream convolutional network to a temporal context is restricted, it is unsuitable for modeling long-time span structures. Videos are one dimension higher than images. Therefore, some scholars optimize the two-dimensional convolutional network into a three-dimensional convolutional network for video image processing tasks.

Ji et al. [46] uses a 3D convolution kernel to extract spatiotemporal features of video data and obtain motion information of the video stream. The model has achieved good performance in the application scenarios of human behavior estimation in airport surveillance videos. Nevertheless, it remains the disadvantage that the 3D convolutional network has a large amount of calculation, which affects the algorithm efficiency. In the work of Wang et al. [27,46], the Openpose model is used to estimate poses of human bodies in videos, and spatiotemporal maps of the key points of human bodies are constructed. The spatiotemporal map convolutional network (ST-GCN) aims to extract spatiotemporal features of human bodies’ key points from continuous video frames. Then, the features are used for video action classification to achieve better human motion estimation performance.

However, although these methods have achieved good results, they are still limited to pure image-based pose estimation. It indicates that these methods will not work well in poor light, so the application range is difficult to broaden.

### 2.2. Sensors-Based Human Pose Estimation

People are constantly exploring the cross-modal human body positioning. By the gyroscope sensor in the bracelet, we can monitor a person’s steps and heartbeat. Cui et al. [47] pointed out that they can detect the number of people and objects in a room by using ultra-wideband radar. The number of people can be predicted by comparing extracted effective information about the radar reflection wave on the obstacle with the sensor data for detecting human bodies.

Khan et al. [48] proposed a human activity estimation method based on acceleration sensors. In some cases, two identification schemes have been established. The first is a low-level solution, which uses statistical signal functions and artificial neural networks (ANN) to identify the current state of behavior, including static, transitional, or dynamic. The second is an advanced scheme, which uses autoregressive (AR) modeling of acceleration signals, and then combines AR coefficients with the signal amplitude area and tilt angle to form an enhanced feature vector. Then, through linear discriminant analysis and artificial neural network, the obtained feature vectors are processed to realize estimation of specific human activities.

### 2.3. WiFi-Based Human Pose Estimation

Wigest [28] performs pose estimation by analyzing rising and falling edges of the signal change represented by the signal strength received by WiFi in different actions. For a single access point and three access points, the estimation accuracy rates reach 87.5% and 96%, respectively. Compared with received signal strength indicator (RSSI), CSI is a fine-grained value at the physical layer. It provides channel estimation for each sub-carrier of each transmission link and reflects the multipath effect caused by small-scale fading and micro-motion. In the work of Wang et al. [29], the CSI-SPEED model proposed by the CARM system quantifies the relationship between CSI amplitude changes and human motion speed and provides a model basis for subsequent research. However, its behavior extraction algorithm is imperfect, and the time complexity of this estimation method is high.

WiHear [27] utilizes CSI changes caused by lip movements with a special directional antenna gain and introduces it into the contour of mouth movements by using the local multipath effect and wavelet packet transform. It can solve the problem of micro-movement detection and achieve a predefined range for words. The average detection accuracy of no more than six words spoken by a single person is 91%, but it is not ideal in signal noise reduction, so strong directional antennas can only be used to reduce noise and improve estimation accuracy. Similarly, Wang et al. [49] proposed a Wi-Alarm system, which ignores the data preprocessing process and uses the support vector machine (SVM) to directly extract the original CSI amplitude mean and variance from human perception as features. Although it can significantly save the calculation cost, the extracted features are not accurate enough for original CSI data to be susceptible to interference from the external environment. The CSI cannot be fully utilized under limited time-domain statistical features. It eventually leads to a system crash and makes estimation accuracy restricted.

Overall, like most WiFi-based human body estimation networks, these methods can only perform rough human body positioning or crowd counting [34,50,51,52,53,54,55,56], but still cannot achieve a fine-grained estimation of the entire human body pose.

## 3. Methodology

### 3.1. Overview

CSI-former aims to realize WiFi-based pose estimation via a teacher–student network: a teacher network estimates human pose in videos by Alphapose, a student network learns human pose from CSI by Performer and convolutional neural network.

Alphapose: Alphapose refers to a traditional two-step frame network for image-based human pose estimation. It achieves high-performance image-based pose estimation via the human detection framework YOlOv3 [57] and the innovative regional multi-person poses estimation framework RMPE [11]. Since Alphapose has shown excellent performance on many public datasets, it is suitable for the teacher network of CSI-former. Thus, Alphapose is used as the ground truth tagger of CSI-former to identify 18 skeleton key points (nose, neck, shoulders, elbows, wrists, hips, knees, ankles, eyes, ears) from human images. Finally, the output of Alphapose is used as the poses ground truth to train the student network.

CSI: Channel State Information (CSI) refers to wireless state information obtained via Orthogonal Frequency Division Multiplexing (OFDM). OFDM converts the high-speed serial data stream into 30 low-speed parallel sub-data streams by decomposing the original channel into 30 mutually orthogonal sub-channels and modulates these sub-data streams to the orthogonal sub-channels for propagation. Each sub-data stream is called a sub-carrier. The amplitude and phase information of all sub-carriers constitute CSI. On this basis, the open-source tools [23] can obtain CSI via characteristics of wireless multipath propagation and analyze its state changes to analyze the changes of the surrounding environment. It indicates that pure CSI can also realize the pose estimation of surrounding human movements.

The original CSI is captured by a three-antenna WiFi transmitter and a three-antenna receiver. The transmitter continuously broadcasts WiFi signals to the outside. When human bodies of different poses pass by, the receiver receives changed wireless signals and parses them into a tensor of m × 30 × 3 × 3 size. Where m represents the number of WiFi packets received, 30 represents the number of wireless sub-carriers, and 3 × 3 represents a 3 × 3 array composed of three transmitting antennas and three receiving antennas.

Unfortunately, it is impossible to annotate real human poses with pure CSI. Thus, we use a camera parallel to the WiFi transmitting antenna to capture human pose images. Then, the captured images are processed by Alphapose of the teacher network to generate annotation information of human poses. Finally, under the supervision of the teacher work, the student network learns pose estimation from CSI.

### 3.2. Attention-Guided DeNoising

**Figure 1:**
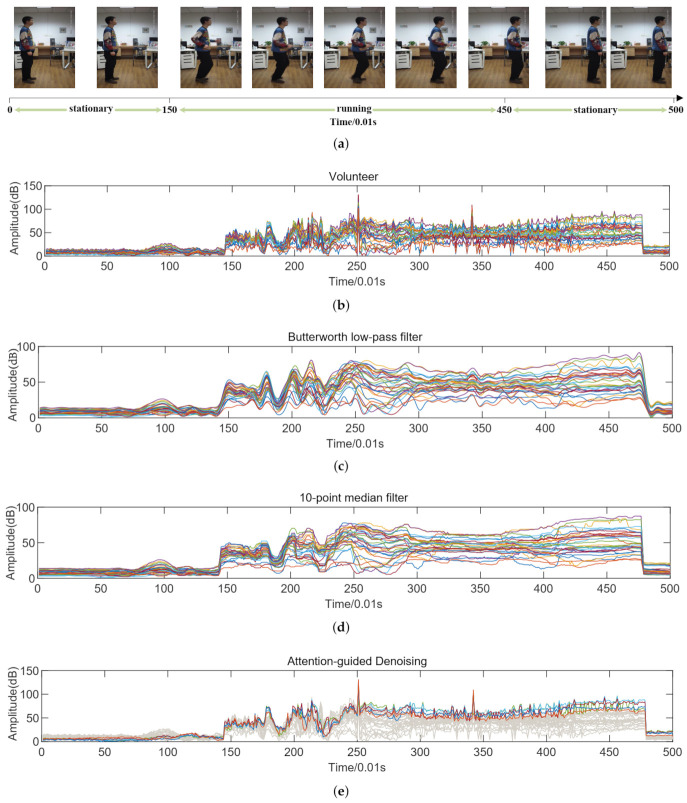
(**a**) records the images of a volunteer from stationary to running to stationary. (**b**) records the corresponding CSI of (**a**) in the time stamp. (**c**,**d**) represents the CSI after Butterworth low-pass filter and ten-pint median filter respectively. The highlighted parts of (**e**) are sub-carriers with richer features and more worthy of attention. The gray parts are sub-carriers that CSI-former tends to pay less attention to.

Inevitably, it tends to generate noise due to environmental influences during the collection of CSI. Under the impact of noise, the performance of the CSI-based pose estimation is bound to be restricted without effective denoising methods. For exploring denoising methods, it is significant to analyze the original CSI. As shown in Figure 1a, it records the images of a volunteer that performs actions after standing still for about 1.5 s. Figure 1b records the corresponding CSI at the timestamp. Since the work of Wang et al. [58] demonstrates that the effect of noise on different sub-carriers is highly correlated, it can be seen in Figure 1b that the amplitudes of all sub-carriers are changing very similarly at the same time when the volunteer is stationary. Nevertheless, when the volunteer performs actions, different sub-carriers have inconsistent changes in amplitude. It indicates that these essential pose feature information in CSI cannot be concealed by noise.

Moreover, Wang et al. [58] also demonstrate that traditional low-pass filters or median filters tend to achieve less-than-satisfactory performance in CSI denoising. As shown in Figure 1, we use the Butterworth low-pass filter that has a sampling rate of 1000 samples per second, and the ten-point median filter to denoise the CSI of Figure 1b respectively. It can be seen that compared to the original CSI, the CSI after filtering becomes very smooth. In addition, even during volunteer activities, the amplitude change of CSI is almost negligible in every small range. It indicates that the hidden pose features of CSI are filtered too. Undoubtedly, it will bring difficulties to the feature extraction of the network.

Inspired by the fact that the pose features cannot be concealed by noise and traditional filters have a poor performance in CSI denoising. It is reasonable to design a new network that can greatly dilute the influence of noise by paying more attention to the pose features. To this end, we propose an attention-guided denoising method (ADN) by CSI-former. Specifically, during the network training, the proposed CSI-former allocates more attention of the network to the sub-carriers that are more sensitive to poses through the parameter updating of the multi-head attention allocation algorithm. As Figure 1e shows, CSI-former pays more attention to these most sensitive sub-carriers that have more pose features and ignores the other sub-carriers with fewer features but much noise. Through the effective distribution of the attention, more pose features are extracted and noise is diluted. Since the sub-carriers are orthogonal to each other and all contain pose features, CSI-former can efficiently allocate attention without losing information.

In addition to ADN, we use multi-frame CSI to align a single-frame image to further dilute the possible impact of noise. In the system settings, the sampling rate of CSI is 100 Hz and the camera’s imaging frequency is 20 Hz. Through the synchronization of timestamps, every five CSI frames are aligned to one image frame. That is, for human body pose annotation information in each image frame, there is a corresponding CSI tensor with the size of 5 × 30 × 3 × 3.

### 3.3. Teacher Network: Alphapose

As the teacher network of CSI-former, Alphapose includes a two-step framework. First, a human body detector is used to form a human body detection box via the input images. After that, a pose estimation network will estimate the pose in the box. The pose skeleton points are finally output as annotation of the teacher network to the student network.

The collected data includes pose images $I_{t}$ (·) and CSI $C_{t}$ (·), aligned by timestamp t∈(0,m). The original pose images $I_{t}$ (·) are processed through the teacher network Alphapose to obtain pose annotation $P_{t}$ (·), which is a 3 × 18 matrix composed of 18 skeleton key points coordinates (x,y) and their confidence c:

(1) $$\begin{array}{c} P_{t}{\left(x,y,c\right)}^{\left(3\times 18\right)}=Alphapose\left(I_{t}\left(\cdot \right)\right),t\in \left(0,m\right). \end{array}$$

**Figure 2:**
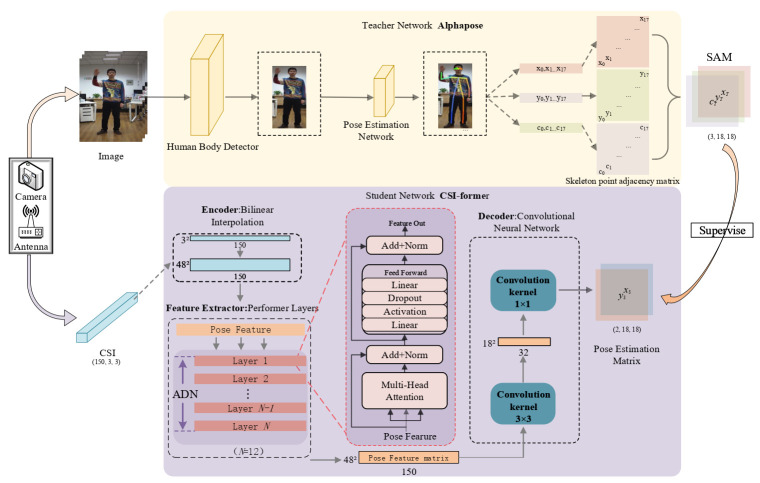
The Alphapose-based teacher network is a two-step framework to analyze the input image and extract 18 skeleton point coordinates to form the SAM. The student network CSI-former is composed of the attention mechanism and convolutional neural network to extract a pose estimation matrix that is adapted to the SAM size from the CSI. Under the supervision of the teacher network, the student network continuously optimizes learning until it achieves the ability of CSI-based pose estimation.

With the original pose annotation $P_{t}$ (·) from the teacher network, the student network needs to realize regression learning of 18 skeleton points of the human body. However, many previous works have demonstrated that it is easy to overfit and lose generalization by simply returning to 18 skeleton points [59]. Therefore, it is necessary to add the skeleton-point adjacency matrix (SAM) as the regular term. As shown in Figure 2, SAM consists of a 3 × 18 × 18 matrix ($x_{i,j}^{\prime }$, $y_{i,j}^{\prime }$, $c_{i,j}^{\prime }$), (i,j∈[1,2,3⋯,18]), where (x,y,c) represents coordinates of skeleton points and theirs confidence. Thus, SAM is a matrix obtained by two-dimensional expansion of ($x_{i},y_{i},c_{i}$), i∈[1,2,3⋯,18] in which *x* and *y* generation rules are the same:

(2) $$\begin{array}{c} x_{i,j}^{\prime }=\left\{\begin{array}{cc} x_{i}-x_{j}, & i\neq j;\\ x_{i}, & i=j. \end{array}c_{i,j}^{\prime }=\left\{\begin{array}{cc} c_{i}\times c_{j}, & i\neq j;\\ c_{i}, & i=j. \end{array}\right.\right. \end{array}$$

SAM enhances the generalization ability of the network via taking relative displacement between skeleton points as an additional constraint. However, it also greatly increases the number of parameters that the network needs to return, and most of the attention is still needs to be paid to the regression of SAM diagonal value. Therefore, the application of the attention mechanism can significantly enhance the performance of the entire network.

Inspired by the SAM, the original pose annotation $P_{t}$ (·) will be expanded to obtain a label matrix with a size of 3 × 18 × 18 as the poses ground truth, which is called $G_{t}$ (·):

(3) $$\begin{array}{c} G_{t}{\left(x^{\prime },y^{\prime },c^{\prime }\right)}^{\left(3\times 18\times 18\right)}=SAM\left(P_{t}\left(\cdot \right)\right) \end{array}$$

Apart from images, the parsed CSI C${}_{t}$ (·) is a tensor with a size of 30 × 3 × 3. Since the image sampling rate and the CSI sampling rate is 20 Hz and 100 Hz respectively, every five C${}_{t}$ (·) will be aligned with one $G_{t}$ (·) through alignment of the time stream. Thus, original WiFi data W${}_{t}$ (·) with a size of 5 × 30 × 3 × 3 for the student network is obtained:

(4) $$\begin{array}{c} W_{t}{\left(\cdot \right)}^{\left(frames\times 30\times 3\times 3\right)}=\sum_{k=t}^{t+4}C_{k}\left(\cdot \right),frames=5 \end{array}$$

### 3.4. Student Network: CSI-Former

The student network is composed of Performer and Convolutional Neural Network. The multi-head attention mechanism makes the student network improve the ability to extract pose features from CSI while learning annotations from the teacher network. Therefore, the student network includes three parts: encoder, feature extractor, and decoder.

Encoder: The encoder is developed to encode the original input $W_{t}$ to adapt to feature extraction. First, the input $W_{t}\in R^{5\times 30\times 3\times 3}$ data size is reshaped to $R^{150\times 3\times 3}$, which makes it correspond to data dimension of the teacher network so that makes convenient for network learning:

(5) $$\begin{array}{c} W_{t}{\left(\cdot \right)}^{\left(5\times 30\times 3\times 3\right)}\to W_{t}^{\prime }{\left(\cdot \right)}^{\left(150\times 3\times 3\right)} \end{array}$$

Then, the encoder directly uses bilinear interpolation to perform preliminary up-sampling on it, and expand it to $R^{150\times 18\times 18}$:

(6) $$\begin{array}{c} W_{t}{\left(\cdot \right)}^{\left(150\times 18\times 18\right)}=Bilinear\left(W_{t}^{\prime }\left(\cdot \right)\right) \end{array}$$

The encoder can not only magnify the features of original data for easy extraction but also make it well adapted to the size of 18 skeleton points annotation from the teacher network.

Attention-BasedFeatureExtractor: Powerful feature extractor can better extract the feature information in the encoded data. Traditional feature extractors are composed of convolutional neural networks or pure ResNet [60]. However, these architectures always perform the same analysis on all input data instead of paying more attention to the more useful information, which limits the performance of the network.

Unlike the traditional methods, CSI-former uses a multi-layer composite attention-based Performer as the feature extractor (PAFE). The PAFE can make the student network pay more attention to those information-rich CSI inputs to realize efficient feature extraction. In addition, considering that the network needs to pay more attention to diagonal elements while taking into account non-diagonal elements when learning SAM, the PAFE can help realize reasonable distribution of attention. After a series of parameter stripping comparison tests, a stack of 12-layers Performer was finally selected. After the encoding matrix $W_{t}\in R^{150\times 18\times 18}$ was extracted by Performer, the feature matrix $F_{t}\in R^{150\times 18\times 18}$ with the same size as the encoding matrix is output:

(7) $$\begin{array}{c} F_{t}{\left(\cdot \right)}^{\left(150\times 18\times 18\right)}=PAFE\left(W_{t}{\left(\cdot \right)}^{\left(150\times 18\times 18\right)}\right) \end{array}$$

Decoder: The decoder is developed to decode the extracted feature matrix $F_{t}$ (·) to match label information $G_{t}$ (·). To achieve this, CSI-former uses the convolutional neural network with a two-layers architecture to be the decoder. The feature matrix $F_{t}\in R^{150\times 18\times 18}$ will go through a convolutional layer firstly. In the layer, the 3 × 3 convolution kernel is used to initially release characteristic information as $F_{t}\in R^{32\times 18\times 18}$, which is followed by the BatchNorm layer and the ReLu layer. Then, the decoder selects a 1 × 1 convolution kernel to completely releases characteristic information $S_{t}\in R^{2\times 18\times 18}$, which contains pose coordinate information estimated by the student network. Finally, L2 norm loss calculation is performed under the supervision of the teacher network:

(8) $$\begin{array}{c} \begin{array}{c} S_{t}{\left(x,y^{\prime }\right)}^{\left(2\times 18\times 18\right)}=Decoder\left(F_{t}\left(\cdot \right)\right)\\ Loss=L2(G_{t}{\left(x^{\prime },y^{\prime },c^{\prime }\right)}^{\left(3\times 18\times 18\right)},S_{t}{\left(x,y^{\prime }\right)}^{\left(2\times 18\times 18\right)}) \end{array} \end{array}$$

Finally, the gradient backpropagation is used to continuously optimize the student network until CSI-former can independently estimate human poses. It should be noted that the trained CSI-former can estimate any individual pose without an entire pose sequence. That is, CSI-former is trained by every single pose rather than ranking different poses.

### 3.5. Loss Calculation

Since CSI-former implements regression learning, the L2 norm loss function which is commonly used in regression learning is suitable for the network [61]. The loss function needs to calculate loss between the output $S_{t}\left(\cdot \right)$ of the student network and the ground truth $G_{t}\left(\cdot \right)$. In addition, the confidence information $c_{i}$ of $G_{t}\left(\cdot \right)$ indicates its relevance to real poses, so it is necessary to take it into account when defining loss function. Finally, the loss function is defined as follows:

(9) $$L=\sum_{i=0}^{17}G^{c_{i}}*\left({∥S^{x_{i}}-G^{x_{i}}∥}_{2}^{2}+{∥S^{y_{i}}-G^{y_{i}}∥}_{2}^{2}\right),$$

where ${∥\cdot ∥}_{2}^{2}$ represents L2 loss calculation; $G^{x_{i}},G^{y_{i}}$ and $S^{x_{i}},S^{y_{i}}$ represents the ground truth and model prediction value of the *i*-th pose respectively. $G^{c_{i}}$ represents confidence of the *i*-th skeleton point.

## 4. Experiments

### 4.1. Data Collection

To establish a rational and persuasive data set under real scenarios, we invited 12 volunteers of different heights and weights to perform multi-action activities indoors while simultaneously using WiFi devices and cameras to capture their wireless status information and images. Each volunteer did 12 different actions (bend, circle, crouch, jump, pull, push, run, sit down, stand up, throw, walk, wave) under guidance. Each action has a period of five seconds and repeats ten times. Since we cannot control the sampling time to exactly 5 s, the actual duration of each action is between 5 and 6 s. Moreover, our camera sampling rate is set to 20 Hz, thus the number of images per action is as follows:

(10) $$\begin{array}{c} N_{min}=12\times 5\times 10\times 20=12000,\\ N_{max}=12\times 6\times 10\times 20=14400, \end{array}$$

**Figure 3:**
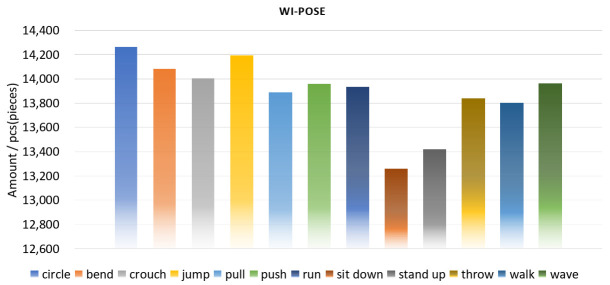
Wi-Pose includes 12 actions with a total of 166,600 data, and the amount of data for each action is roughly equal. We analyze and organize the collected raw data, and remove some data with unclear images or inaccurate skeleton points. Wi-Pose is finally composed of the remaining clear images, its corresponding CSI, and skeleton point annotation.

We aligned and split the collected videos and CSI through time stamps, and put each video frame and its corresponding CSI data in a formatted file. Therefore, there are about 12,000 to 14,400 data for each action. The data number of Wi-Pose is 166,600 and the specific number of each action is shown in Figure 3. After analysis and sorting, the Wi-Pose that is composed of human images, its corresponding CSI, and skeleton point annotations was finally established.

For data division in the experiment, 80% of the collected data were used for the training set and the remaining 20% for the testing set. The number of the training set and the testing set are 132,847 and 33,753, respectively.

### 4.2. Model Evaluation

For evaluating the model, the percentage of correct key points (PCK) is one of the most persuasive evaluation algorithms for human pose key point detection models. It evaluates the CSI-former by calculating the ratio of the normalized distance between 18 detected key points and their corresponding ground truth that is less than the set threshold. The reference of normalized distance is developed via the torso diameter as follows:

(11) $$TD_{k}=\sqrt[{2}]{{\left(G_{k}^{RS_{x}}-G_{k}^{LH_{x}}\right)}^{2}+{\left(G_{k}^{RS_{y}}-G_{k}^{LH_{y}}\right)}^{2}},$$

where $TD_{k}$ is the diameter of the *k*-th body’s torso, $G_{k}^{RS}$ and $G_{k}^{LH}$ are the ground truth of the *k*-th body’s right shoulder and left Hip coordinates respectively. The calculated Euclidean distance between these two points is approximated as torso diameter. The specific algorithm of PCK is as follows:

(12) $$PCK_{k_{i}}@a_{j}=\frac{1}{N}\sum_{i=1}^{N}\delta \left(\frac{∥S_{k}^{i}-G_{k}^{i}{∥}_{2}^{2}}{TD_{k}}\leq a_{j}\right),$$

where $a_{j}$ is the *j*-th threshold of the algorithm, and the above formula $PCK_{k_{i}}@a_{j}$ represents the PCK value of the *i*-th skeleton key point of the *k*-th human pose under the threshold, i=[1,2,⋯,18]. The value of $\delta \left(\cdot \right)$ refers to a boolean value whose value is one when the inequality in parentheses is true and otherwise zero. $S_{k}^{i}$ and $G_{k}^{i}$ respectively represent the coordinates of the *i*-th joint point of the *k*-th person identified by the model and the ground truth of the joint point. After the normalization calculation and the discrimination of $\delta \left(\cdot \right)$, the model finally obtains the average predicted PCK of each skeleton point of the testing set.

The threshold of PCK is usually set between 5 and 50. It should be noted that different thresholds are the evaluation standard of the model at different scales. Therefore, in this paper, we set the thresholds to various values between 5 and 50 to demonstrate the performance of our model from different evaluation scales. The larger the threshold is, the wider the error margin of skeleton point estimation is allowed. Thus, increasing the threshold may lead to higher PCK. However, lower thresholds represent more strict evaluation criteria, meaning lower thresholds can better demonstrate the model’s performance.

### 4.3. Implementation Details

**Figure 4:**
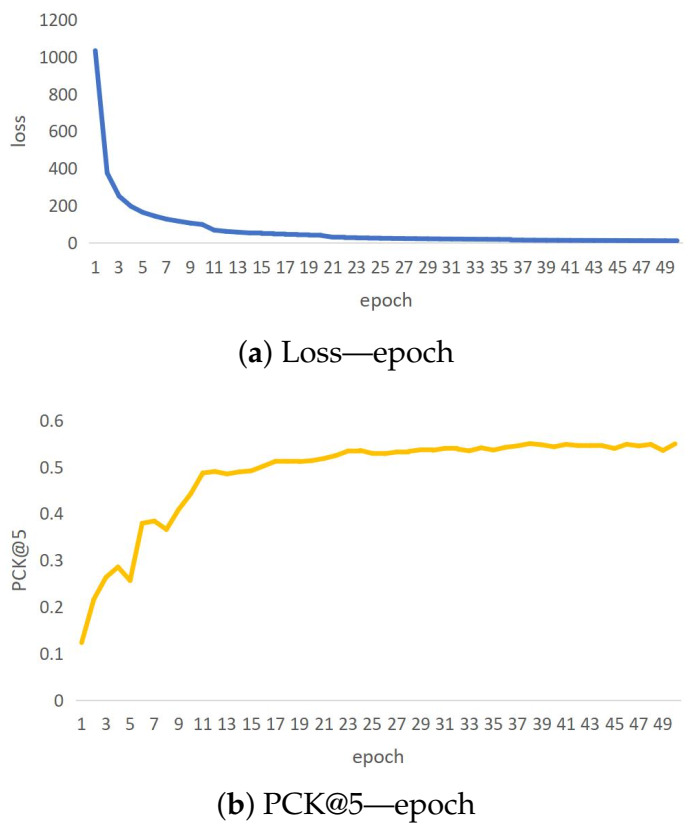
CSI-former stops training when the loss converges and the PCK reaches the highest level. This goal can usually be achieved by training 50 epochs. Thus, CSI-former has been trained for a total of 50 epochs. The trend graph of loss and PCK during training is shown in (**a**) and (**b**) respectively.

CSI-former is implemented by Pytorch 1.7 and optimized by Adam optimizer. The batch size and epochs-number are eight and 50, respectively. The initial learning rate is set to 0.005 and is halved every ten epochs in the first 20 epochs and halved every 15 epochs in the last 30 epochs. We usually choose the model weights of the epoch which has the minimum loss. The epoch with minimum loss is generally between 45 to 50. The specific training details of the loss and the PCK are shown in Figure 4.

### 4.4. Ablation Study

In this section, we perform a series of ablation studies to analyze the impact of Performer layers on the performance of CSI-former. Under the strictly same training condition, four, eight, and twelve Performer layers were developed respectively to the feature extractor of CSI-former. The final result shown in Table 1 indicates that CSI-former with more layers of Performer tends to achieve higher PCK@5 on almost all skeleton points estimation. It demonstrates that under the same conditions, the more layers of Performer, the better performance of CSI-former. Thus, CSI-former is finally proposed via 12 layers stacked attention-based Performer. To further investigate the performance of CSI-former, we calculate the PCK of each skeleton point estimated via CSI-former by different thresholds of five, ten, twenty, thirty, forty, fifty, respectively. As shown in Table 2, it indicates CSI-former achieves excellent performance on the estimation of human poses.

### 4.5. Model Comparison

CSI-former uses 12-layers superimposed Performer as the feature extractor of the network. In order to prove the effectiveness of the multi-head attention mechanism, we compared the effects of CSI-former with pose estimation network using 16-layers superimposed Resnet as the feature extractor. After training models under the same experimental condition, we evaluated the two models with the same testing set. As shown in Table 3, it records the PCK@5 of 12 skeleton points in the testing set that are estimated by CSI-former and Resnet respectively. It is obvious that CSI-former achieves more superior performance over the Resnet on the estimation of ten different skeleton points and achieves a 2.7% increase in average PCK. The experimental result demonstrates that the multi-head attention mechanism significantly improves the pose estimation performance of the network.

**Figure 5:**
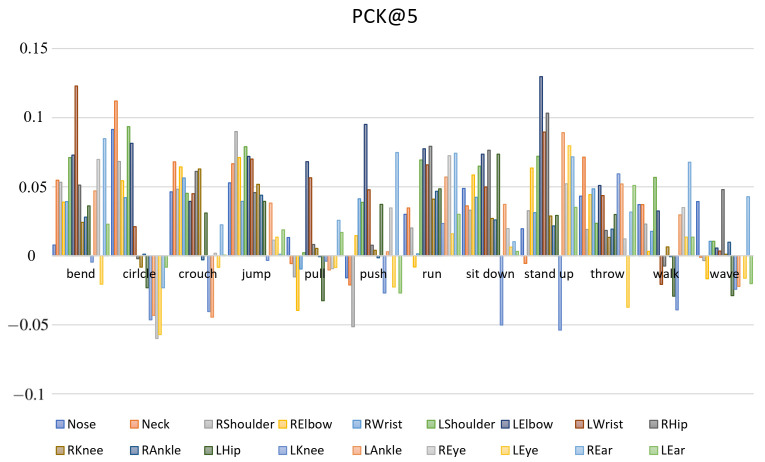
For each action, the vertical axis records the per-joint changes in the PCK@5 results of the proposed CSI-former relative to Resnet. The positive value means that CSI-former’s prediction accuracy for the skeleton joint is higher than Resnet.

Additionally, Figure 5 separately records the difference between the PCK results by CSI-former and Resnet for 18 skeleton points of different actions. It can be seen that the estimation performance of skeleton points of most actions (such as bend, jump, and run) has been greatly improved by CSI-former over Resnet. It indicates that through the biased allocation of attention by ADN, CSI-former significantly improves the estimation performance of most poses at the cost of reducing the accuracy of some poses. In other words, CSI-former successfully pays more attention to those sub-carriers that are sensitive to most actions and pays less attention to these subcarriers that are sensitive to individual actions to improve the overall predictive ability of the model.

### 4.6. Experimental Result

**Figure 6:**
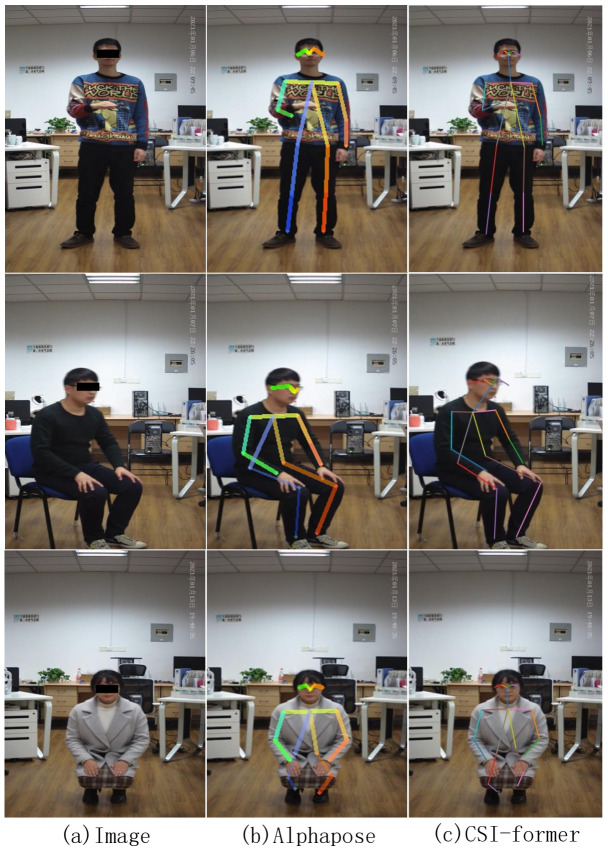
(**a**) records the original human images of three volunteers. (**b**,**c**) records the pose estimation results of the three original human images by Alphapose and CSI-former, respectively. Each pose estimation draws 18 key skeleton points of the human body and connects these skeleton points in the order of the human body on the original image. It should be noted that the estimation of CSI-former on some skeleton points is better than Alphapose.

**Figure 7:**
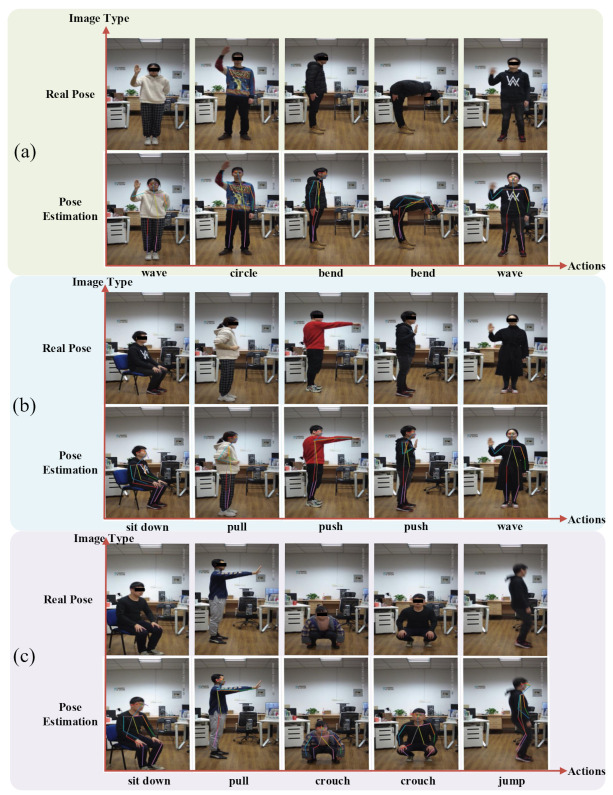
Wi-Pose includes different actions of persons with different heights and weights. (**a**–**c**) are three groups of images of volunteers selected randomly. The first row of each group shows the real poses of the volunteers, and the corresponding second row shows the pose estimation results of CSI-former. The results demonstrate that CSI-former achieves superior performance on WiFi-based pose estimation.

To observe the performance of CSI-former more directly, we estimate the poses of the testing set via CSI-former. Then, the estimated poses of CSI-former are drawn on the original images and compared with the pose estimation of the Alphapose-based teacher network, as shown in Figure 6. It can be observed that CSI-former achieves better performance over Alphapose on some skeleton points estimation. Additionally, we record the poses of volunteers with different heights and weights estimated by CSI-former in Figure 7. The experimental results demonstrate that the attention mechanism can significantly improve the performance of the WiFi-based pose estimation network and CSI-former achieves a superior pose estimation performance.

### 4.7. Result Discussion

As shown in the above sections, we finalized CSI-former through a series of ablation studies. We compare CSI-former with existing Resnet-based networks in Table 3, and CSI-former achieves higher PCK. As seen in Figure 6 and Figure 7, CSI-former has achieved satisfactory performance on WiFi-based human pose estimation.

Nevertheless, due to the constraints of time cost and experimental environment, CSI-former is trained by 12 activities performed by 12 volunteers. It indicates that the pose estimation results may have deviations when CSI-former is applied to other complex activities. Thus, we will expand more poses in more environments to our dataset Wi-Pose in future research. Since there is no publicly available dataset for WiFi-based human pose estimation currently, we have published Wi-Pose to facilitate future research. Moreover, we will continue to study new algorithms to improve CSI-former and enable it to estimate multi-person poses.

## 5. Conclusions

In this paper, we propose a WiFi-based pose estimation network CSI-former. Compared to traditional image-based pose estimation methods, CSI-former overcomes the disadvantages of cameras that cannot work in the dark and glare by using WiFi. Moreover, the WiFi-based method can solve the privacy concerns in human pose estimation well.

We design the architecture of CSI-former by 12 layers of Performer with multi-head attention to make the network pay more attention to the information that includes more pose features. The experimental results in Table 3 show that the PCK@5 of CSI-former achieves 0.5505, which is higher than the existing Resnet-based method whose PCK@5 is 0.5231. It demonstrates that CSI-former has better pose estimation performance than the existing Resnet-based method.

In addition, we successfully establish a novel WiFi-based human pose estimation dataset Wi-Pose and have published Wi-Pose to promote future research. CSI-former achieves state-of-the-art performance on Wi-Pose. Our future work will focus on further improving the structure of CSI-former to obtain better WiFi-based pose estimation capabilities. 

## Figures and Tables

**Table 1:** Comparisons between different Perormer layers in CSI-former. The bold value of each row is the optimal value.

Skeleton-Point		PCK@5	
	**Layers = 4**	**Layers = 8**	**Layers = 12**
Nose	0.4870	0.5598	**0.5615**
Neck	0.5106	**0.5915**	0.5910
RShoulder	0.4993	0.5747	**0.5787**
RElbow	0.3668	0.4318	**0.4387**
RWrist	0.2795	0.3504	**0.3534**
LShoulder	0.4515	**0.5455**	0.5432
LElbow	0.3171	0.4338	**0.4484**
LWrist	0.2876	0.3742	**0.4041**
RHip	0.4976	0.5955	**0.6171**
RKnee	0.5785	0.6649	**0.7093**
RAnkle	0.5971	0.7139	**0.7422**
LHip	0.4700	0.5890	**0.6050**
LKnee	0.5430	0.6509	**0.6674**
LAnkle	0.5276	0.6619	**0.6904**
REye	0.4872	0.5661	**0.5918**
LEye	0.3440	0.5023	**0.5240**
REar	0.4983	0.5749	**0.6027**
LEar	0.2150	**0.2456**	0.2391
Average	0.4421	0.5348	**0.5505**

**Table 2:** 12 Performer layers are used. The capitalized L and R refer to Left and Right, respectively. The bold value of each row is the optimal value.

Skeleton-Point	PCK@5	PCK@10	PCK@20	PCK@30	PCK@40	PCK@50
Nose	0.5615	0.6543	0.7403	0.7857	0.8193	**0.8421**
Neck	0.5910	0.6839	0.7706	0.8177	0.8481	**0.8708**
RShoulder	0.5787	0.6769	0.7618	0.8097	0.8411	**0.8663**
RElbow	0.4387	0.5583	0.6729	0.7353	0.7791	**0.8120**
RWrist	0.3534	0.4709	0.5960	0.6660	0.7149	**0.7547**
LShoulder	0.5432	0.6533	0.7583	0.8122	0.8436	**0.8679**
LElbow	0.4484	0.5611	0.6756	0.7436	0.7908	**0.8254**
LWrist	0.4041	0.5145	0.6247	0.6967	0.7462	**0.7807**
RHip	0.6171	0.7119	0.8001	0.8452	0.8730	**0.8920**
RKnee	0.7093	0.7905	0.8606	0.8930	0.9163	**0.9322**
RAnkle	0.7422	0.8108	0.8606	0.8902	0.9060	**0.9174**
LHip	0.6050	0.7118	0.7943	0.8398	0.8682	**0.8881**
LKnee	0.6674	0.7644	0.8364	0.8710	0.8932	**0.9080**
LAnkle	0.6904	0.7583	0.8166	0.8475	0.8685	**0.7807**
REye	0.5918	0.6772	0.7550	0.8021	0.8316	**0.8453**
LEye	0.5240	0.6188	0.7145	0.7687	0.8089	**0.8357**
REar	0.6027	0.6902	0.7668	0.8108	0.8444	**0.8643**
LEar	0.2391	0.2944	0.3874	0.4610	0.5169	**0.5560**
Average	0.5505	0.6445	0.7329	0.7831	0.8172	**0.8417**

**Table 3:** Comparisons between attention-based and attention-free methods. The bold value of each row is the optimal value.

Skeleton-Point	PCK@5	
	Resnet	CSI-Former
Nose	0.5271	**0.5615**
Neck	0.5539	**0.5910**
RShoulder	0.5525	**0.5787**
RElbow	0.4099	**0.4387**
RWrist	0.3234	**0.3534**
LShoulder	0.4910	**0.5432**
LElbow	0.3818	**0.4484**
LWrist	0.3546	**0.4041**
RHip	0.5760	**0.6171**
RKnee	0.6880	**0.7093**
RAnkle	0.7263	**0.7422**
LHip	0.5875	**0.6050**
LKnee	**0.6850**	0.6674
LAnkle	0.6706	**0.6904**
REye	0.5715	**0.5918**
LEye	**0.5273**	0.5240
REar	0.5620	**0.6027**
LEar	0.2275	**0.2391**
Average	0.5231	**0.5505**

## Data Availability

Not applicable.
